# Factors influencing anterior/low anterior resection syndrome after rectal or sigmoid resections

**DOI:** 10.3906/sag-2007-145

**Published:** 2021-04-30

**Authors:** Sami BENLİ, Tahsin ÇOLAK, Mehmet Özgür TÜRKMENOĞLU

**Affiliations:** 1 Department of Surgery, Division of Colorectal Surgery, Faculty of Medicine, Mersin University, Mersin Turkey

**Keywords:** Bowel dysfunction, low anterior resection syndrome, rectal cancer, quality of life

## Abstract

**Background/aim:**

Sphincter-preserving surgery is one of the main goals in the treatment of rectal cancer because it improves the quality of life (QoL). However, some patients may experience disrupted symptoms called anterior or low anterior resection syndrome (LARS). This study was designed to evaluate the frequency and influencing factors of LARS in patients who underwent sigmoid or rectal resection.

**Materials and methods:**

In this retrospective, clinical study, patients who underwent rectal or sigmoid resection and anastomosis due to any benign and malignant reasons were evaluated in terms of LARS between January 2010 and November 2019 at Medical Faculty Hospital of Mersin University. The frequency and severity of LARS were determined by using a standard scale. Furthermore, influencing factors including lesion localization, operation, the proximity of anastomosis to the anal verge, creation of stoma, chemotherapy, and radiotherapy application were investigated.

**Results:**

Out of a total of 550 patients, 276 were included in this study. The major LARS incidence was found as 27.2%. Very low anterior resection (VLAR) (OR = 42.40 (95% CI [11.14–161.36], P < 0.0001), protective ileostomy (OR = 12.83 (95% CI [6.58–25.0], P < 0.0001), end colostomy (OR = 8.55 (95% CI [1.36–53.61], P = 0.022), receiving chemotherapy (OR = 3.08 (95% CI [1.71–5.53], P < 0.0001), and radiotherapy (OR = 2.51 (95% CI [1.38–4.57], P = 0.003) and the ROC analysis showed that creating an anastomosis placed at most 8.5 cm from the anal verge was found to be a major influencing factor on LARS (P < 0.05).

**Conclusions:**

LARS may frequently occur in patients who have undergone rectal resection. In this study, the most important factors influencing LARS were found to be the proximity of anastomosis to the anal canal and creating a protective stoma. Receiving chemoradiotherapy also plays an important role in LARS.

## 1. Introduction

Colorectal cancers are among the most common cancer types in Europe and one-third of the cases are rectal cancer [1]. Also, colorectal cancers constitute the third most common cancer in Turkey, with approximately 40% of the cases being rectal cancer [2]. Advances in surgical techniques and chemoradiotherapy result in a better local control of rectal cancer, which provides an opportunity for sphincter-sparing surgery especially in mid or lower rectal cancer. Thus, sphincter-sparing surgery with total mesorectal excision (TME) becomes a gold standard in the treatment of rectal malignancies due to improving Quality of Life (QoL) [3–6]. However, sphincter-sparing surgery does not always improve QoL and sometimes causes problems with anorectal and urinary systems; moreover, sexual dysfunctions may develop after sphincter-preserving rectal surgery. Patients who have undergone low anterior resection (LAR) due to rectal cancer experience major defecation problems as a result of weakening the reservoir and neurosensory capacity of the rectum [7]. Incontinence, fecal frequency, and urgency may be observed among these defecation problems, and all of these symptoms are called anterior or low anterior resection syndrome (LARS) [7,8]. In various studies, the prevalence of LARS varies between 25%–80% depending on the definition of the syndrome, the frequency, and the intensity of follow-up [9–12]. Various risk factors have been identified in the development of LARS such as age, female gender, surgical technique, prolonged temporary stoma condition, neoadjuvant and adjuvant chemotherapy or/and radiotherapy (CRT), and postoperative complications [13,14]. Furthermore, some studies reported that neoadjuvant RT [15] and adjuvant RT [16] are among the most important factors. On the other hand, some studies stated that low anastomosis level is the most important risk factor [12].

In this study, we evaluated the factors influencing LARS and the prevalence of LARS by using a standard LARS scale in patients who underwent sigmoid or rectal resection and anastomosis in a single colorectal center.

## 2. Materials and methods

This study was designed retrospectively for the purpose of evaluating the frequency of LARS and the influencing factors in patients who underwent anterior resection, low anterior resection, and very low anterior resection for benign and malignant reasons at the Department of Colorectal Surgery ofthe Medical Faculty at Mersin University,between January 2010 and November 2019. All the procedures were performed by the same surgical team in the Training and Research Hospital of the university,where annually more than 100 cases of colorectal procedures are performed, approximately half of which include rectal procedures. All parts of the study and access to the electronic medical records were approved by the Human Research Ethics Committee of Mersin University, Mersin, Turkey. All the participants consented to a structured interview wherein they completed a questionnaire to assess their defecation functions. The inclusion criteria included age over 18 years and sigmoid or rectal resection and anastomosis for either benign or malignant reasons. Those who met these criteria were invited to participate in the study. Only patients who had at least a 12-month follow-up period, who had protective stoma reversed, and who completed their adjuvant treatments were included the study. Protective ileostomy was created in all the patients who had rectal anastomosis located at least 6 cm from the anal verge or anastomotic difficulties or preference of the attending surgeon. Also, stoma closing was accepted to be early if stoma was reversed in less than 6 months or to be late if reversed in more than 6 months. 

The exclusion criteria included patients who left for another center for their follow-up or who were lost during the follow-up period, patients who had impaired cognitive functions, patients whose information was not fully accessible from the hospital information management system, and patients who could not be able to have their stoma closed or had local recurrence. 

**Figure 1:**
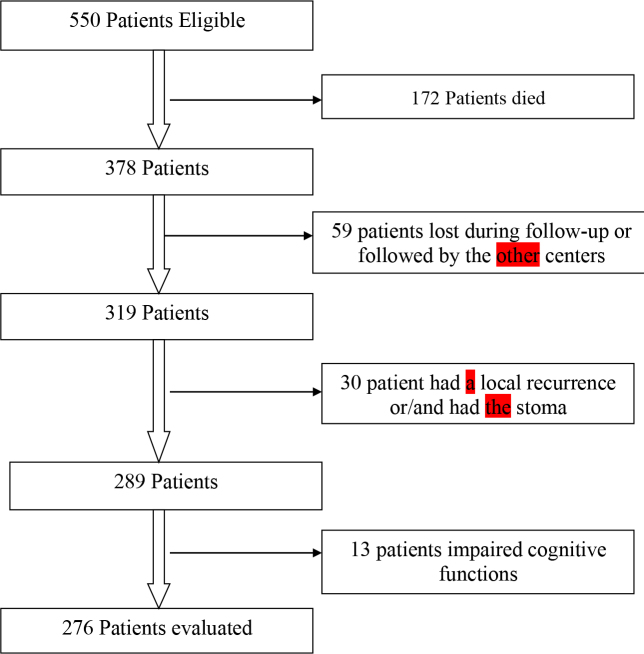
Flow chart of the study.

A total of 550 patients were found to be eligible for this study. Of these, 172 patients died, 59 patients continued treatment in another center for their follow-up or discontinued to apply to our center during the follow-up period, 30 patients were not suitable to close the stoma, and 13 patients who had impaired cognitive functions were excluded from the study. Finally, the remaining 276 patients were evaluated (Figure1).

The demographic data, the localization of the tumor, the type of operation, the level of anastomosis, the presence of a protective stoma and the time to close the stoma, receiving neoadjuvant/adjuvant chemotherapy or/and radiotherapy, and the pathology reports were obtained using the electronic data record system of the hospital. The types of operation were described as anterior resection (AR) if the anastomosis level was above the pelvic peritoneum, low anterior resection (LAR) if the anastomosis level was below the pelvic peritoneum but higher than the anal canal, and very low anterior resection (VLAR) if the coloanal anastomosis or intersphincteric resection was performed.

The calculation of the patients’ LARS scores was performed after interviewing the patients by phone or during routine outpatient clinic controls. A standard LARS questionnaire was applied to the patients during the interview, and five questions (gas incontinence, liquid-solid incontinence, fecal frequency, urgency, and 1-h bowel opening frequency) were asked, and then scored between 0 and 42 points in the questionnaire [8]. The patients were divided into three groups depending on the LARS score. These groups were non-LARS (0–20), minor LARS (21–29), and major LARS (30–42).

## 3. Statistical analysis

 The analysis of the data was performed with Statistica Version 13.5.0.17 (TIBCO Software Inc., Palo Alto, CA, USA; 2017). The mean ± standard deviation or ranges ​​from descriptive statistics for age, and the frequency and percentage values ​​were used for categorical variables. Also, the proportion of the patients choosing each response in the individual LARS scale items was similarly compared. Using clinical judgment and the research to date, the potential risk factors for severe LARS (major LARS vs. minor or no LARS) were tested using multiple logistic regression analysis: age (median vs. > median age), sex (female vs. male), the distance of the tumor to the anal verge, additional surgery (yes vs. no), the type of operations, ASA score, and the total number of collected lymph nodes and stoma closure.

*χ* *2* tests were used for the comparison of categorical variables. P values of the Pearson chi-square coefficient were taken into consideration. Spearman’s rho correlation coefficient was used for the correlation between two continuous variables. 

Multinomial logistic regression analysis, one of the multivariate statistical methods, was used to determine the variables that may have significant risk factors on the LARS score. Multinominal logistic regression analysis was performed for variables that could be significant risk factors according to the P < 0.25 rule on the three-category LARS score. The major LARS category was selected as the reference category. The significance of the independent variables in the model was tested with the P value of the Wald statistic. Odds ratios (OR) and 95% confidence intervals (CI) were calculated. An odds ratio with a confidence interval not including “1” was considered statistically significant. The “non-LARS” reference category was selected. 

In addition, LARS categories were reorganized as “no LARS” and “LARS” for the ROC analysis. According to the new LARS groups, the optimum cut-off value for the anastomosis level and the area under the curve (AUC) were determined by the ROC analysis. The Youden’s index was used to find the best cut point for anastomosis level. The statistical significance level (P) was considered as <0.05 for all the comparisons.

## 4. Results

The incidence of LARS was calculated as 36.3% (27.2% of them being major LARS and 9.1% of them being minor LARS). The mean age was 60.2 ± 12.9 years and the male/female ratio was 172/104. The ASA scores of the patients were recorded as 30.4% ASA 1, 48.6% ASA 2, 17.8% ASA 3, and 3.3% ASA 4.

There were no significant differences in terms of age (<65, >65), sex (male or female), ASA scores (ASA1, ASA2, ASA3, and ASA4 ), stage (stage 1, stage 2, stage 3, and stage 4), the time of closing stoma (early closing <6 months, versus late closing >6 months), additional surgery (TAH + BSO, liver resection, small bowel resection, omentectomy, cholecystectomy, and splenectomy), and the number of collected lymph nodes. These factorswere not found to be effective on the prevalence of LARS (P = 0.14, P = 0.69, P = 0.56, P = 0.69, P = 0.07, P = 0.74, and P = 0.82 respectively). These results were summarized in Tables 1 and 2.

**Table 1:** Preoperative clinicopathological features of the patients.

	Total	Non-LARS		Minor LARS		Major LARS		P value
		Cases	(%)	Cases	(%)	Cases	(%)	
All	276	176	63.8	25	9.1	75	27.2	
Sex								0.687
· Male	172	113	64.2	15	60.0	44	58.7	
· Female	104	63	35.8	10	40.0	31	41.3	
Age (years)								0.475
· >65	113	107	60.8	16	64.4	40	53.3	
· 18–65	163	69	39.2	9	36.0	35	46.7	
ASA score								0.364
· ASA 1	84	51	29	9	36	24	32	
· ASA 2	134	93	52.8	11	44.0	30	40.0	
· ASA 3	49	27	15.3	5	20.0	17	22.7	
· ASA 4	9	5	2.4	0	0.0	4	3.3	
Lesion location								<0.0001
· Rectum	152	73	41.5	18	72.0	61	81.3	
· Sigmoid	124	103	58.5	7	28.0	14	18.7	
Malignancy								<0.0001
· Yes	233	142	80.7	20	80.0	71	94.7	
· No	43	34	19.3	5	20.0	4	5.3	

LARS: anterior/low anterior resection syndrome. The data was given as the number of patients and percentiles in parenthesis.

**Table 2:** Postoperative clinicopathological features of the patients and their difference between groups with LARS.

	Total	Non-LARS		Minor LARS		Major LARS		P Value
	Cases	Cases	(%)	Cases	(%)	Cases	(%)	
All	276	176	63.8	25	9.1	75	27.2	
Type of operation								<0.0001
· VLAR	26	3	1.7	5	20.0	18	24.0	
· LAR	122	67	38.1	13	52.0	42	56.0	
· AR	128	106	60.2	7	28.0	15	20.0	
Ostomy state								<0.0001
· Ileostomy	81	20	11.4	16	64.0	45	60.0	
· Colostomy	8	2	1.1	3	12.0	3	4.0	
· No stoma	187	154	87.5	6	24.0	27	36.0	
AJCC stage								0.981
· Stage 1	49	31	22.1	4	20.0	14	20.0	
· Stage 2	75	48	34.3	6	30.0	21	30.0	
· Stage 3	81	47	33.6	8	40.0	26	37.1	
· Stage 4	25	14	10.0	2	10.0	9	12.9	
Chemotherapy								<0.0001
· Yes	148	80	45.5	14	56.0	54	72.0	
· No	128	96	54.5	11	44.0	21	28.0	
Radiotherapy								<0.0001
· Yes	176	95	54.0	16	64.0	53	74.7	
· No	95	81	46.0	9	36.0	19	25.3	
Synchronous surgery								0.130
· Yes	44	29	8.0	7	16.0	7	1.3	
· No	233	147	83.5	18	72.0	68	90.7	

VLAR: very low anterior resection, LAR: low anterior resection, AR: anterior resection.The data was given as the number of patients and percentiles in parenthesis.

A total of 138 stomas were closed in the included patients. Of these, 50 were closed with in the first 6 months after their operations and they were accepted as early stoma closure, whereas 88 of them were closed later than the first 6 months and those were accepted as late stoma closure. No significant differences were observed among the LARS groups when compared in terms of early vs. late stoma closure (P = 0.742).

The types of operations were VLAR in 9.4%, AR in 44.2%, and LAR in 46.4% of the patients. Major LARS was observed in 69.2% of the VLAR patients, in 34.4% of the LAR patients, and 11.7% of the AR patients. It was shown that the increase in the incidence of LARS was directly proportional to the proximity of the anastomosis to the anal canal and these differences between the groups were found to be significant (P < 0.0001). 

Chemotherapy was also a significant effect on the LARS incidence (OR = 3.08 (95% CI [1.71–5.53], P < 0.0001). A significant relationship was seen between minor LARS and neoadjuvant CT (P = 0.0001), whereas adjuvant CT was found to be associated with major LARS (P = 0.032).

When the effects of neoadjuvant and adjuvant radiotherapy on LARS categories were evaluated, neoadjuvant RT was found to be related to minor LARS (P = 0.039), whereas adjuvant RT administration significantly increased major LARS (P = 0.012).

The patients were operated for malignant reasons in 84.4% of the cases andfor benign reasons in 15.6% of the cases. Major LARS was found in 9.3% of the patients operated for benign reasons and in 30.4% operated for malignant reasons (P = 0.016). Although malign causes were detected in 84.4% of the patients with major LARS, only 15.6% of the patients with major LARS were operated due to benign reasons.

When the patients were divided according to the localization of the lesions, 55.1% of the lesions were located in the rectum and 44.9% were in the sigmoid. While the rate of major LARS was 66.1% in those with a lesion in the rectum, it was found to be 11.2% in those with a sigmoid lesion, and a statistically significant difference was found between the lesion localization and LARS (P < 0.0001). There was a statistically significant relationship between the rectum and major LARS. According to the multinomial logistic regression analysis, rectal localization was 6.11 times more likely to have major LARS than those with sigmoid (OR = 6.14 (95% CI [3.19–11.82]).

In this study, 53.6% of the patients received CT, and 60.5% of the patients received RT. Among the patients who received CT, 72% had major LARS. This difference was statistically significant when compared to the proportion of non-LARS with major LARS patients. The multinomial logistic regression analysis showed that major LARS was 3.08 times more likely to occur in patients who received CT than those who did not (OR = 3.08 (95% CI [1.71–5.53]).There was a statistically significant relationship between RT and major LARS, and major LARS was 2.51 times more likely to be observed in patients that received RT than those who did not (OR = 2.51 (95% CI [1.38–4.57]).

When the relationship between stoma condition and LARS was evaluated, major LARS was observed in 60.0% of the patients with protective ileostomy and in 36.0% of the patients without a stoma. This difference was statistically significant. The probability of major LARS was 12.83 times (OR = 12.83 (95% CI [6.58–25.0]) higher in the patients with protective ileostomy compared to those without a stoma. Also, according to calculations, this difference was 8.55 times more likely to be seen in major LARS patients with end colostomy when compared to the patients without a stoma (OR = 8.55 (95% CI [1.36–53.61]).

In the comparison of the relationship between LARS and the type of operation due to lesion localization, major LARS was seen in 69.2% of the patients with VLAR, while it was seen in 34.4% of the patients with LAR and in 11.7% of the patients with AR. The probability of developing major LARS was calculated as 42.40 times higher in the patients with VLAR than those with AR when compared to the prevalence of LARS in the patients with VLAR and AR (OR = 42.40 (95% CI (11.14–161.36). The patients who had LAR surgery were also more likely to experience major LARS than the patients with AR due to proximal rectal or sigmoidal lesions (OR = 4.43 (95% CI [2.28–8.60]) (Table 3).

**Table 3:** The density of factors influencing LARS on the multinomial logistic regression analysis.

Parameters	OR	95% CI	P value
Malignant reasons	0.23	0.08–0.68	0.008
RT	2.51	1.38–4.57	0.003
CT	3.08	1.71–5.53	<0.0001
LAR	4.43	2.28–8.60	0.029
End colostomy	8.55	1.36–53.61	0.022
Temporary ileostomy	12.83	6.58–25	<0.0001
VLAR	42.40	11.14–161.36	<0.0001

RT: radiotherapy, CT: chemotherapy, OR: odds ratio, CI: confidence interval.

**Figure 2:**
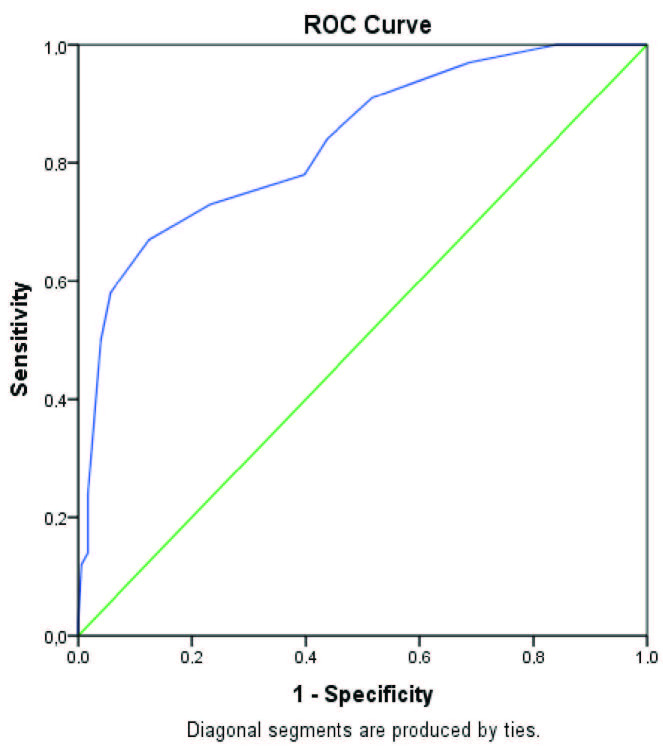
The receiver operating characteristics (ROC) curve between sensitivity and 1-specifity of anastomotic level in defining major LARS. Area under ROC curve = 0.834.

The median levels of the anastomoses in the groups with and without LARS showed a statistically significant difference. The median of the group with LARS (6.5 cm) was lower than the median of the non-LARS group (12 cm). As a result of the analysis of the ROC curve for the anastomosis level, the area under the ROC curve (AUC) value was calculated as 0.834. The AUC value was statistically significant (P < 0.05), and the anastomosis level was quite high in distinguishing between LARS and non-LARS. According to the Youden’s index, the cut-off point was found to be 8.5 cm proximal to the anal verge. Accordingly, those who had an anastomosis placed ≤8.5 cm to the anal verge demonstrated a significantly higher risk for developing LARS. The sensitivity value was calculated as 67% and the specificity value as 87.5% for the 8.5 cm threshold (Figure 2).

## 5. Discussion

The prevalence of LARS was revealed to be between 25%–80% of the patients who underwent sphincter sparing surgery [17], but it is not yet clear which factors affect the occurrence of LARS. Although LARS has a significant incidence in patients who have had a sphincter-sparing operation due to the rectum tumor, it is still often overlooked by surgeons. Furthermore, the densities of the effects of the related factors are not clear either. Some researchers reported that patients younger than 64 years, TME, anastomotic leakage, postoperative chemotherapy or radiotherapy, protective ileostomy, and female gender are considered as risk factors in anterior or low anterior resection syndrome [18–20]. Changes in bowel compliance, decreased neo-rectum reservoir capacity, and weakened sphincters are also considered as other possible causes [15,21].

In our study, we found that the critical anastomotic level for LARS is <8.5 cm proximal to the anal verge. The critical threshold was found different in some studies. In the study conducted by Miacci et al. [22], this value was found to be 6.5 cm; however, other studies found this value as 5 cm [23]. Though the threshold values given in other studies are different, all of these studies have confirmed that low or very low anastomosis might have resulted with severe LARS.

Contrary to the literature, in this study, age was not found to be effective in any form of LARS. Furthermore, age was also not found to be a factor affecting the incidence of LARS in elderly patients. A similar debatable result was stated in the sex factor in the literature as well. In this study, female gender was not an effective factor in the occurrence of LARS. In the study conducted by Kupsch et al. [23], being under the age of 64 was associated with LARS, whereas Croese et al. [24] did not show the effect of over 65 years of age and female gender on LARS. This study was in accordance with the study conducted by Juul et al. [25], which revealed that age and sex were not effective on LARS. 

In our study, the effects of chemotherapy or radiotherapy, lesion localization, and protective stoma were found to be statistically significant in accordance with other studies [8,13,14]. In addition to the literature, this study demonstrated that CT or RT might result in minor LARS if given neoadjuvant therapy, while these might result inmajor LARS when given adjuvant therapy. These findings lead us to reconsider the timing of administration of chemotherapy again if we are suspecting LARS.

However, the proven important causes affecting LARS syndrome include RT, CT, protective stoma, low anastomosis level, and postoperative complications. Previous studies indicated that the stoma closing time is an important factor in the occurrence of LARS and they speculated that changing the muscular and mucosal layers of the colon affects the structure and function of the neorectum. Early closing of the stoma reverses this process and less structural and functional impairment develops with a lower incidence of LARS. This study confirms that protective ileostomy increases the LARS incidence approximately 13 times. However,the stoma closing time was not found to be effective on LARS. These results might be dependent on our comparison when considering that the first 6 months would be too long a period to be accepted as an early period.

In many studies, the reasons affecting LARS in common are similar. These include CRT, distal lesion, and protective stoma. However, it has not been clearly shown which of these factors have greater effects on LARS, because most patients with distal lesions are given CT and RT and a protective stoma is created. In today’s conditions, evaluating these factors separately does not seem appropriate in terms of disrupting the treatment of patients. 

To minimize these errors, the multinomial logistic regression analysis, a multivariate statistical method, was used in this study to determine the factors that have significant effects on the incidence of LARS. In light of these results, the type of operation and the anastomosis level were observed as the most important factors related to the LARS development. This study indicated that the patients who underwent VLAR had 43 times higher risk of facing LAR than those who underwent AR. In the presence of these important findings, we performed a further analysis to determine the level of anastomosis cut-off point and we found that a proximity of 8.5 cm to the anal verge is the peak level of anastomosis, and anastomosis below this level is more likely to trigger LARS. In addition, the multinomial logistic regression analysis showed that the patients who underwent an operation for the malign causes experienced LARS slightly more than for the benign reasons. Also, rectal resections and postoperative RT increased the incidence of LARS.

Although it seems that the most effective method in the treatment and prevention of LARS is colostomy opening, the effect of opening colostomy for LARS on the quality of life of the patient is still controversial [26].

## 6. Conclusion

The incidence of LARS was found to be 36.3% in this study. CT and RT might be associated with minor LARS when neoadjuvant therapy is applied, whereas major LARS was usually associated with an adjuvant therapy application. In addition, when the patients who have undergone rectal resection for the malignant disease have a slight risk, patients with a protective stoma have a moderate risk of experiencing LARS. Furthermore, the type of rectal operation, especially VLAR, is the most important factor for LARS. Also, making an anastomosis placed at most 8.5 cm from the anal verge is found to be another important factor in the occurrence of LARS. On the other hand, contrary to the literature, age, sex, the stage of the disease, and the collected lymph nodes were not effective on the LARS incidence.

## Funding

The authors received no financial support for the research and/or authorship of this article.
